# Multiphase and multiparameter MRI-based radiomics for prediction of tumor response to neoadjuvant therapy in locally advanced rectal cancer

**DOI:** 10.1186/s13014-023-02368-4

**Published:** 2023-10-31

**Authors:** Hongyan Huang, Lujun Han, Jianbo Guo, Yanyu Zhang, Shiwei Lin, Shengli Chen, Xiaoshan Lin, Caixue Cheng, Zheng Guo, Yingwei Qiu

**Affiliations:** 1 Department of Radiology, The Third Affiliated Hospital of Guangzhou Medical University, Guangzhou Medical University, Duobao AVE 56, Liwan District, Guangzhou, People’s Republic of China; 2 Department of Radiology, Huazhong University of Science and Technology Union Shenzhen Hospital, Taoyuan Road #89, Nanshan District, Shenzhen, 518000 People’s Republic of China; 3 Department of Medical Imaging, Sun Yat-Sen University Cancer Center, 651 Dongfeng Road East, Guangzhou, 510060 People’s Republic of China; 4 Department of Radiology, Meizhou People’s Hospital, No. 63 Huangtang Road, Meizhou, 514000 China; 5 Department of Hematology and Oncology, International Cancer Center, Shenzhen Key Laboratory of Precision Medicine for Hematological Malignancies, Shenzhen University General Hospital, Shenzhen University Clinical Medical Academy, Shenzhen University Health Science Center, Xueyuan AVE 1098, Nanshan District, Shenzhen, 518000 Guangdong People’s Republic of China

**Keywords:** Locally advanced rectal cancer, Neoadjuvant therapy, Tumor response, MRI, Radiomics

## Abstract

**Background:**

To develop and validate radiomics models for prediction of tumor response to neoadjuvant therapy (NAT) in patients with locally advanced rectal cancer (LARC) using both pre-NAT and post-NAT multiparameter magnetic resonance imaging (mpMRI).

**Methods:**

In this multicenter study, a total of 563 patients were included from two independent centers. 453 patients from center 1 were split into training and testing cohorts, the remaining 110 from center 2 served as an external validation cohort. Pre-NAT and post-NAT mpMRI was collected for feature extraction. The radiomics models were constructed using machine learning from a training cohort. The accuracy of the models was verified in a testing cohort and an independent external validation cohort. Model performance was evaluated using area under the curve (AUC), sensitivity, specificity, positive predictive value, and negative predictive value.

**Results:**

The model constructed with pre-NAT mpMRI had favorable accuracy for prediction of non-response to NAT in the training cohort (AUC = 0.84), testing cohort (AUC = 0.81), and external validation cohort (AUC = 0.79). The model constructed with both pre-NAT and post-NAT mpMRI had powerful diagnostic value for pathologic complete response in the training cohort (AUC = 0.86), testing cohort (AUC = 0.87), and external validation cohort (AUC = 0.87).

**Conclusions:**

Models constructed with multiphase and multiparameter MRI were able to predict tumor response to NAT with high accuracy and robustness, which may assist in individualized management of LARC.

**Supplementary Information:**

The online version contains supplementary material available at 10.1186/s13014-023-02368-4.

## Introduction

Rectal cancer (RC) is one of the most common malignant tumors in the digestive tract [1], with 70% of cases being locally advanced rectal cancer (LARC) [2]. Preoperative neoadjuvant therapy (NAT) has been considered as a standard treatment regimen for LARC [3]. Nevertheless, some patients do not benefit from preoperative NAT or even show disease progression after NAT [4]. For these patients, a more tailored treatment strategy should be adopted to avoid the unnecessary toxicity of radiation and chemotherapy [5]. Thus, it is critical to identify reliable biomarkers to predict patients with non-response to NAT before its administration for personalized therapy. Approximately 15–27% of patients with LARC achieve pathologic complete response (pCR) after NAT [6]; for these patients, organ-preserving and function-preserving strategies, such as local excision and watch-and-wait, might be an alternative treatment method, which shows no significant difference from radical resection in 3-year overall survival [7]. Meanwhile, radical resection had been reported to have a long-term impact on anorectal, urinary, and sexual function in patients with LARC, which in turn resulted in poor quality of life for these patients [8]. Therefore, it is also crucial to stratify patients with pCR after NAT but before surgical section in order to take a watch-and-wait approach to avoid unnecessary surgery.

Tumor heterogeneity remains the major factor hindering accurate identification of tumor response to NAT [9]. Many studies have attempted to explore serological or genetic biomarkers for early prediction of response to NAT, but robust biomarkers have not yet been found [10, 11]. Magnetic resonance imaging (MRI) is recommended as a noninvasive method for evaluating therapeutic response [12]. Studies have shown that multiparameter MRI (mpMRI) data, such as T2-weighted imaging (T2WI), diffusion-weighted imaging (DWI), and dynamic contrast-enhanced imaging (DCE), possess diverse diagnostic information for assessing therapeutic response to NAT in LARC patients [13–15]. However, their performances are limited by insufficient information and interobserver variability [16]. Therefore, it is necessary to extract more information from image-based features.

Radiomics, as a computer-aided approach to analyzing medical images, could effectively improve accuracy in detection and triage [17], precision diagnosis [18], evaluating treatment response [19], and predicting prognosis [20] by extracting minable features related to tumor heterogeneity [21, 22]. Recently, radiomics has been widely used to predict treatment response and to assess pCR for LARC patients [19, 23, 24]. Using pretreatment mpMRI, Zhou et al. built a radiomics model to identify LARC patients with non-response to NAT. Although their model performed well in their testing group (AUC of 0.77, 95% confidence interval of 0.61–0.94), the generalizability of their model is limited due to the single-center data collection [23]. Based on post-NAT mpMRI, Shin et al. successfully constructed a prediction model for pCR in LARC patients after NAT but before radical resection; they obtained a powerful predictive value in recognizing pCR, but their study was also limited by potential risks of overfitting due to the lack of external independent validation and failing to include pre-NAT MRI data [24], which may provide comprehensive information on tumor heterogeneity [22].

To resolve these limitations, in the present study, we recruited relatively large samples from two independent centers to (1) construct a multiparameter MRI model (Model_NoRes_NAT) with a pre-NAT multiparameter MRI signature to predict non-response to NAT before its administration in LARC patients; (2) build a multiphase and multiparameter MRI model (Model_pCR) based on both pre-NAT and post-NAT MRI data to recognize pCR in LARC patients after NAT but before surgical resection.

## Materials and methods

### Patients

This retrospective study was conducted in accordance with the Declaration of Helsinki and was approved by the local institutional review board. The requirements for informed consent were waived because of the retrospective design.

A total of 704 patients with LARC were retrospectively recruited from two institutions in China, including 539 patients from *BLINDED* (Center 1) and 165 patients from *BLINDED* (Center 2). The detailed inclusion/exclusion criteria are illustrated in Additional file 1: Fig. S1.

## MRI protocol

All patients underwent mpMRI scans within 1 week before NAT and/or within 1 week before surgery (defined as pre- and post-NAT MRI, respectively). MRI sequences included T2WI, DWI, and contrast-enhanced T1-weighted imaging (CE-T1WI). Details in the Additional file 1: Table S1.

**Table 1:** Clinical and treatment characteristics for all patients

	Center 1 (n = 453)	Center 2 (n = 110)
Age (years, mean ± SD) *	55.34 ± 11.12	59.2 ± 10.6
*Sex (%)*		
Male	299 (66.0%)	85 (77.3%)
Female	154 (34.0%)	25 (22.7%)
*Clinical stage (%)*		
II	93 (20.5%)	11 (10.0%)
III	321 (70.9%)	97 (88.2%)
IV	39 (8.6%)	2 (1.8%)
*Pretreatment T stage (%)*		
2	10 (2.2%)	1 (0.9%)
3	269 (59.4%)	51 (46.4%)
4	174 (38.4%)	58 (52.7%)
*Lymph node status (%)*		
LN negative	94 (20.8%)	14 (12.7%)
LN positive	359 (79.2%)	96 (87.3%)
*Location (%)*		
> 10 cm	68 (15.0%)	10 (9.1%)
5–10 cm	235 (51.9%)	41 (37.3%)
< 5 cm	150 (33.1%)	59 (53.6%)
*Pretreatment CEA (%)*		
< 5 (normal)	245 (54.1)	52 (47.3%)
≥ 5 (abnormal)	208 (45.9%)	58 (52.7%)
*Pretreatment CA199 (%)*		
< 39 (normal)	392 (86.5%0	93 (84.5%)
≥ 39 (abnormal)	61 (13.5%)	17 (15.5%)
*Neoadjuvant therapy (%)*		
Neoadjuvant chemoradiotherapy	403 (89.0%)	110 (100.0%)
Neoadjuvant chemotherapy	50 (11.0%)	–
*Mandard grade (%)*		
1	104 (23.0%)	25 (22.7%)
2	125 (27.6%)	31 (28.2%)
3	138 (30.5%)	29 (26.4%)
4	80 (17.7%)	25 (22.7%)
5	6 (1.3%)	–

Unless otherwise specified, data are numbers of patients, with percentages in parentheses. * Data are means ± standard deviations. Clinical stage was based on pretreatment computed tomography of the chest and abdomen and pelvis magnetic resonance imaging, according to the 8th edition of the AJCC Staging Manual. LN status was defined by case. Tumor location was categorized based on distance from the anorectal verge: < 5 cm, 5–10 cm, and > 10 cm. The pretreatment CEA and CA199 level were tested within one week before neoadjuvant therapy. CEA, carcinoembryonic antigen; CA199, carbohydrate antigen-199

## Neoadjuvant therapy

All eligible participants had been previously treated with standard NAT (5-fluorouracil-based chemotherapy orally or intravenously with or without concurrent radiotherapy delivered at 50 Gy/25 times/5 weeks for gross tumor volume and clinical tumor volume 45–46 Gy/25 times/5 weeks). Total mesorectal excision (TME) was performed within 6–8 weeks after completion of NAT.

## Pathological assessment of response

Pathologic response after NAT was reassessed based on resection specimens by an experienced pathologist and was further reviewed by a dedicated gastrointestinal pathologist, both of whom were blinded to the MRI data. Pathologic grading of primary tumor regression was conducted according to the Mandard’s tumor regression grade (TRG) system [25]. According to Mandard’s TRG system, patients were categorized into response to NAT with TRG 1–3 [26], while pCR was defined as the absence of viable tumor cells in the primary tumor (TRG 1) [24].

## MRI assessment

The MRI-based tumor regression grade (mrTRG) was assessed by a radiologist with dedicated experience in rectal cancer imaging based on a five-point system [27]. We defined mrTRG 1 as pCR and 2–5 as non-pCR.

## Tumor segmentation and feature extraction

All radiomics analyses were conducted with the Darwin research platform (https://arxiv.org/abs/2009.00908), which included tumor segmentation, feature extraction, and model construction. Tumor contour was manually delineated slice-by-slice on all sequences and features were extracted by Pyradiomics software. Intra-class correlation coefficients (ICCs) were calculated to evaluate reproducibility of features [28].

## mpMRI-based radiomics model construction for predicting non-response to NAT

Eligible patients from center 1 were randomly split into a training cohort and testing cohort at a ratio of 8:2. Min–max normalization was performed to make features comparable and to obtain the effect of reducing the prediction error and training time. To reduce overfitting, three feature selection steps were performed. First, a two-sample *t* test was used for initial selection; next, the least absolute shrinkage and selection operator (LASSO) algorithm was applied to further select robust and non-redundant features, and the optimal adjustment parameters were determined by tenfold cross-validation; finally, we used a recursive feature selection support vector machine (Ref-SVM) to identify key features. Due to unbalanced sampling between the non-responder and responder groups, the cost-sensitive learning method was utilized to address the balance by strongly penalizing mistakes in the non-responder group [29]. Thereafter, a multiparameter MRI model (Model_NoRes_NAT) was constructed with the selected features for predicting non-responders to NAT using the linear kernel support vector machine algorithm in the training cohort. The performance of the multiparameter MRI model was initially evaluated in the internal testing cohort and then validated in the independent external validation cohort by receiver operating characteristic (ROC) curve analysis. For comparisons, three single-sequence prediction models, which were based on imaging features derived from pre-NAT T2WI (T2WI model), DWI (DWI model), and CE-T1WI (CE model), were also developed using a similar method. The radscore was calculated for all patients using the radiomic score formula derived from the training cohort to verify the classification performance of models.

## Multiphase mpMRI-based radiomics model construction for evaluating pCR

We constructed four different models for prediction of pCR based on features from mrTRG, pre-NAT multiparameter MRI, post-NAT multiparameter MRI alone or in combination of both pre-NAT and post-NAT multiparameter MRI. Firstly, similar feature selection strategies were performed for the pre-NAT and post-NAT multiparameter MRI. Thereafter, a multiphase and multiparameter MRI radiomics model (Model_pCR) was constructed for the noninvasive assessment of pCR using the linear kernel SVM algorithm. Two single phase models (pre-NAT model and post-NAT model), and one mrTRG model (model constructed with mrTRG) were also constructed and for further comparisons. ROC curve analysis was utilized to assess the performance of radiomics models in the testing cohort and in the external independent validation cohort. Concurrently, radscore was also calculated for all patients using the radiomic score formula derived from the training cohort.

## Statistical analysis

ROC curve analysis was used to determine the diagnostic accuracy of models in the training cohort, testing cohort, and external validation cohort. Accuracy (ACC), positive predictive value (PPV), negative predictive value (NPV), sensitivity, specificity, the area under the receiver operating characteristic curve (AUC), and the corresponding 95% confidence interval (CI) were utilized to evaluate predictive performance. The calibration curve detected the goodness of fit between the prediction and the actuality. Decision curve analysis (DCA) represented the clinical net benefits brought by the model. The DeLong test was used to compare AUCs of different models. The *t* test or Mann–Whitney *U* test was used for continuous variables, and the *χ*^2^ test or Fisher test was used for categorical variables. A two-tailed *P*-value of < 0.05 was considered significant. All statistical analysis was conducted with R software (version 3.4.0, http://www.R-project.org) and IBM SPSS Statistics (version 25.0).

## Results

### Clinical characteristics

A total of 563 patients who fulfilled the inclusion criteria (for construction of Model_NoRes_NAT) were finally included in the present study; then, 362 patients from center 1 were included in the training cohort, 91 patients from center 1 were included in the internal testing cohort, and 110 patients from center 2 served as the independent external validation cohort. Out of the 563 patients, 476 fulfilled the inclusion criteria for construction of Model_pCR; a total of 292 patients from center 1 were included in the training cohort, 74 patients from center 1 were included in the internal testing cohort, and 110 patients from center 2 served as the independent external validation cohort. Detailed clinical characteristics of patients are summarized in Table 1 and Additional file 1: Tables S2 and S3.

## Intra- and inter-observer reproducibility evaluation

Satisfactory intra-observer and inter-observer reproducibility was acquired for the tumor segmentation and the extraction of imaging features (ICCs > 0.6).

## Performance of mpMRI radiomics model

**Fig. 1:**
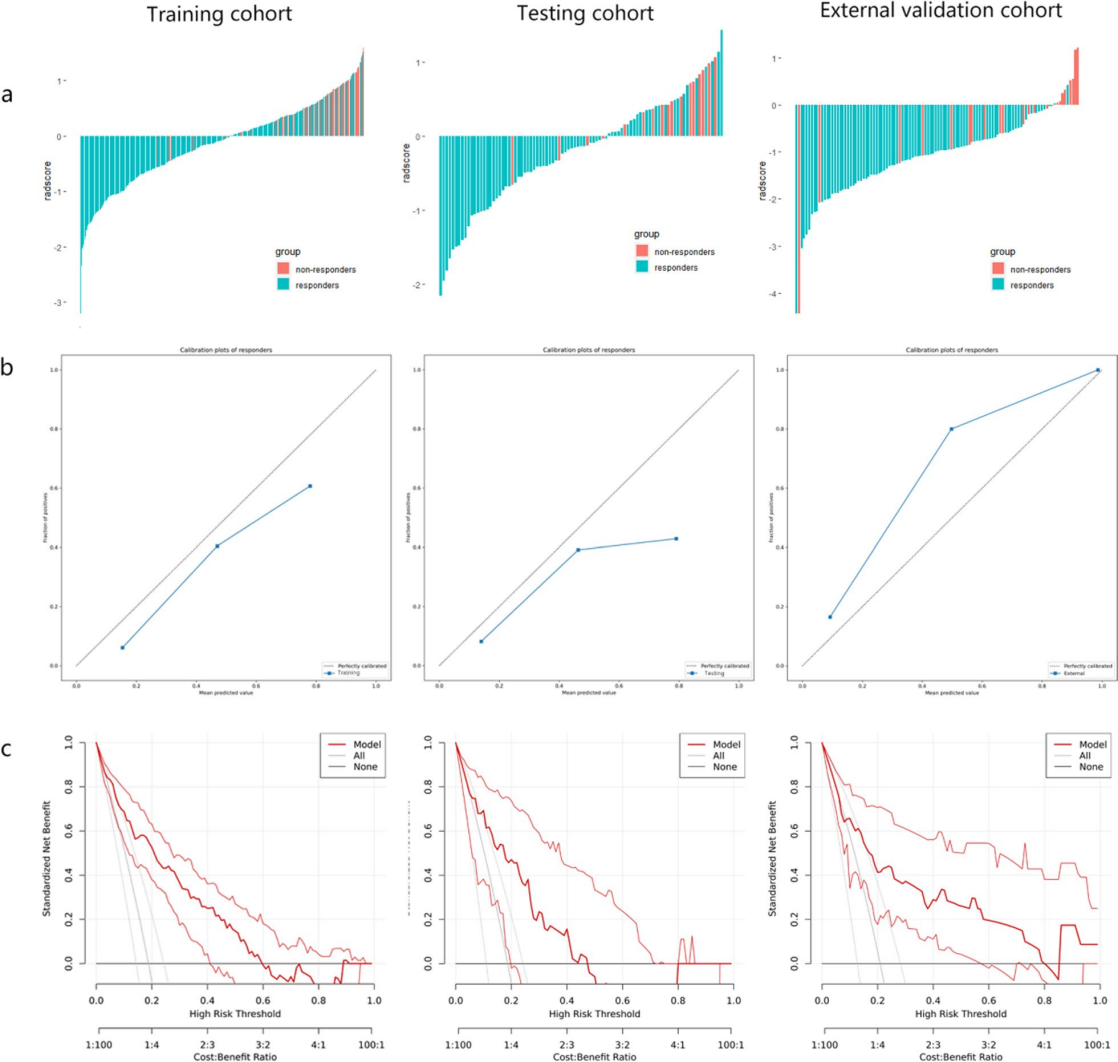
Prediction performance of Model_NoRes_NAT and comparison with single-sequence prediction models. **a** Receiver operating characteristic curve analysis of Model_NoRes_NAT in the training cohort (*n* = 362), internal testing cohort (*n* = 91), and external validation cohort (*n* = 110) for predicting non-response to neoadjuvant therapy. **b**, **c** Receiver operating characteristic curve analysis of Model_NoRes_NAT compared with three single-sequence prediction models (T2WI, DWI, and CE model) in the internal testing cohort (*n* = 91) and the external validation cohort (*n* = 110). T2WI, T2-weighted imaging; DWI, diffusion-weighted imaging; CE, contrast-enhanced T1-weighted imaging; Model_NoRes_NAT, pre-NAT multiparametric magnetic resonance imaging-based radiomics model

**Fig. 2:**
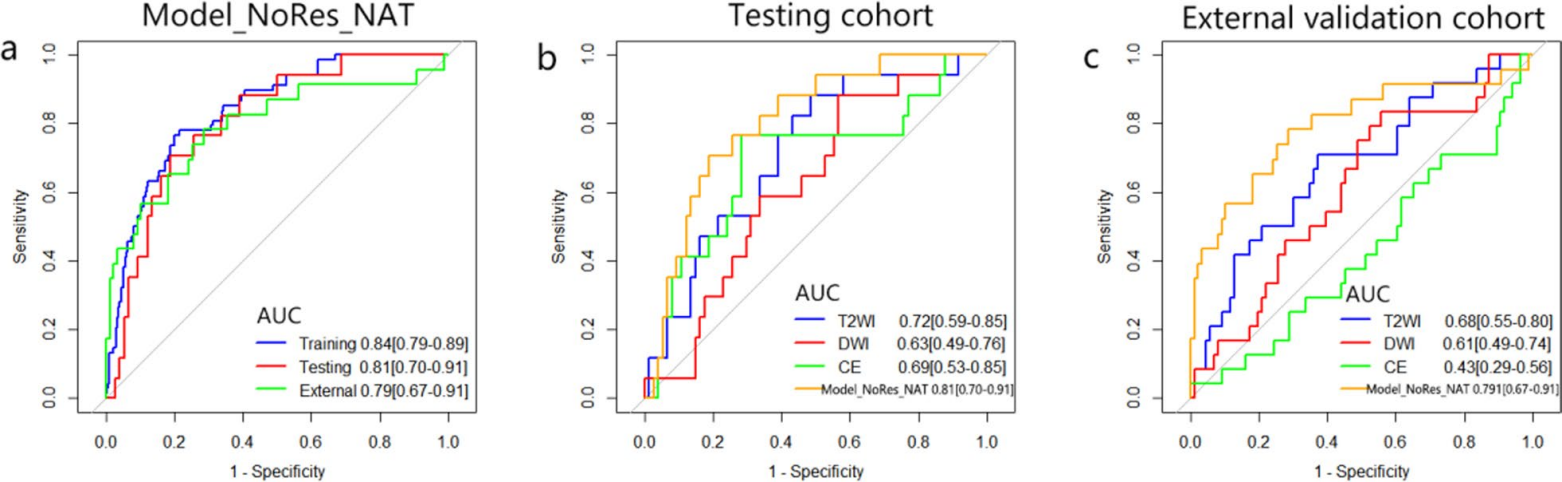
Radscore, calibration curves, and decision curves to predict non-response to neoadjuvant therapy. **a** Radscore for each patient in the training cohort (*n* = 362), internal testing cohort (*n* = 91), and external validation cohort (*n* = 110). **b** Calibration curves for Model_NoRes_NAT in the training cohort, internal testing cohort, and external validation cohort. The dotted reference line indicates perfect calibration. **c** Decision curves for Model_NoRes_NAT in the training cohort, internal testing cohort, and external validation cohort. The y-axis represents the net benefit. The x-axis represents the threshold probability. The red line represents Model_NoRes_NAT. The gray line represents the assumption that all patients showed non-response to neoadjuvant therapy. The black line represents the hypothesis that no patients showed non-response to neoadjuvant therapy. The threshold probability is where the expected benefit of treatment is equal to the expected benefit of avoiding treatment. Model_NoRes_NAT, pre-NAT multiparametric magnetic resonance imaging-based radiomics model

After a strict feature selection strategy, 20 imaging features (including 12 T2WI features, 4 DWI features, and 4 CE-T1WI features) were selected from pre-NAT mpMRI to construct Model_NoRes_NAT for prediction of non-response to NAT (detailed features can be found in Additional file 1: Table S4). As shown in Fig. 1a, Model_NoRes_NAT had good performance in predicting non-response to NAT in the testing cohort (AUC 0.81, 95% CI 0.70–0.91) and independent external validation cohort (AUC 0.79, 95% CI 0.67–0.91). The sensitivity was 70.6, 78.3 and specificity was 79.7, 71.3 for the testing cohort and the external validation cohort, respectively. The NPV (92.2 and 92.5 for testing and external validation cohort, respectively) was relatively superior to the PPV (44.4 and 41.9 for testing and external validation cohort, respectively), which may be due to the disproportionate rate of non-responders in our study population (Table 2). The AUC values of the T2WI, DWI, and CE-T1WI models for predicting non-responders to NAT were 0.72 (95% CI 0.59–0.85), 0.63 (95% CI 0.49–0.76), and 0.69 (95% CI 0.53–0.85), respectively, in the testing cohort and 0.68 (95% CI 0.55–0.80), 0.61 (95% CI 0.49–0.74), and 0.43 (95% CI 0.29–0.56) in the external validation cohort (Fig. 1b–c, Additional file 1: Table S5). Compared with three single-sequence models, Model_NoRes_NAT yielded higher AUC in the prediction of non-responders to NAT in the two validation cohorts (Additional file 1: Table S6). The overall distribution of radscore was obviously different between responders and non-responders in all cohorts (*p* < 0.001), suggesting Model_NoRes_NAT achieved excellent classification performance (Fig. 2a). The calibration curve of the radiomics model presented satisfactory consistency between prediction and observation in all validation cohorts (Fig. 2b). The decision curve indicated that the radiomics model could bring more clinical net benefits for patients in all validation sets (Fig. 2c).

**Table 2:** Performance of the Model_NoRes_NAT and Model_pCR in the prediction of tumor response to neoadjuvant therapy in LARC patients

	AUC (95%)	Sensitivity (95%)	Specificity (95%)	PPV (95%)	NPV (95%)	Accuracy
*Model_NoRes_NAT*						
Training cohort	0.84 [0.79–0.89]	77.9 [66.7–86.2]	78.6 [73.5–82.9]	45.7 [36.9–54.8]	93.9 [90.2–96.3]	0.78
Testing cohort	0.81 [0.70–0.91]	70.6 [46.9–86.7]	79.7 [69.2–87.3]	44.4 [27.6–62.7]	92.2 [83.0–96.6]	0.78
External validation cohort	0.79 [0.67–0.91]	78.3 [58.1–90.3]	71.3 [61.0–79.7]	41.9 [28.4–56.7]	92.5 [83.7–96.8]	0.73
*Model_pCR*						
Training cohort	0.86 [0.81–0.91]	71.2 [60.1–80.4]	85.4 [80.1–89.5]	61.9 [51.2–71.6]	89.9 [85.1–93.3]	0.82
Testing cohort	0.87 [0.76–0.98]	85.0 [64.0–94.8]	83.3 [71.3–91.0]	65.4 [46.2–80.6]	93.8 [83.2–97.9]	0.84
External validation cohort	0.87 [0.78–0.95]	84.0 [65.4–93.6]	81.2 [71.6–88.1]	56.8 [40.9–71.3]	94.5 [86.7–97.9]	0.82

Data in parentheses are 95% CIs. Model_NoRes_NAT is for non-response prediction and Model_pCR is for pathological complete response prediction. AUC, area under the curve; PPV, positive predictive value; NPV, negative predictive value

## Performance of Multiphase mpMRI-based radiomics model

**Fig. 3:**
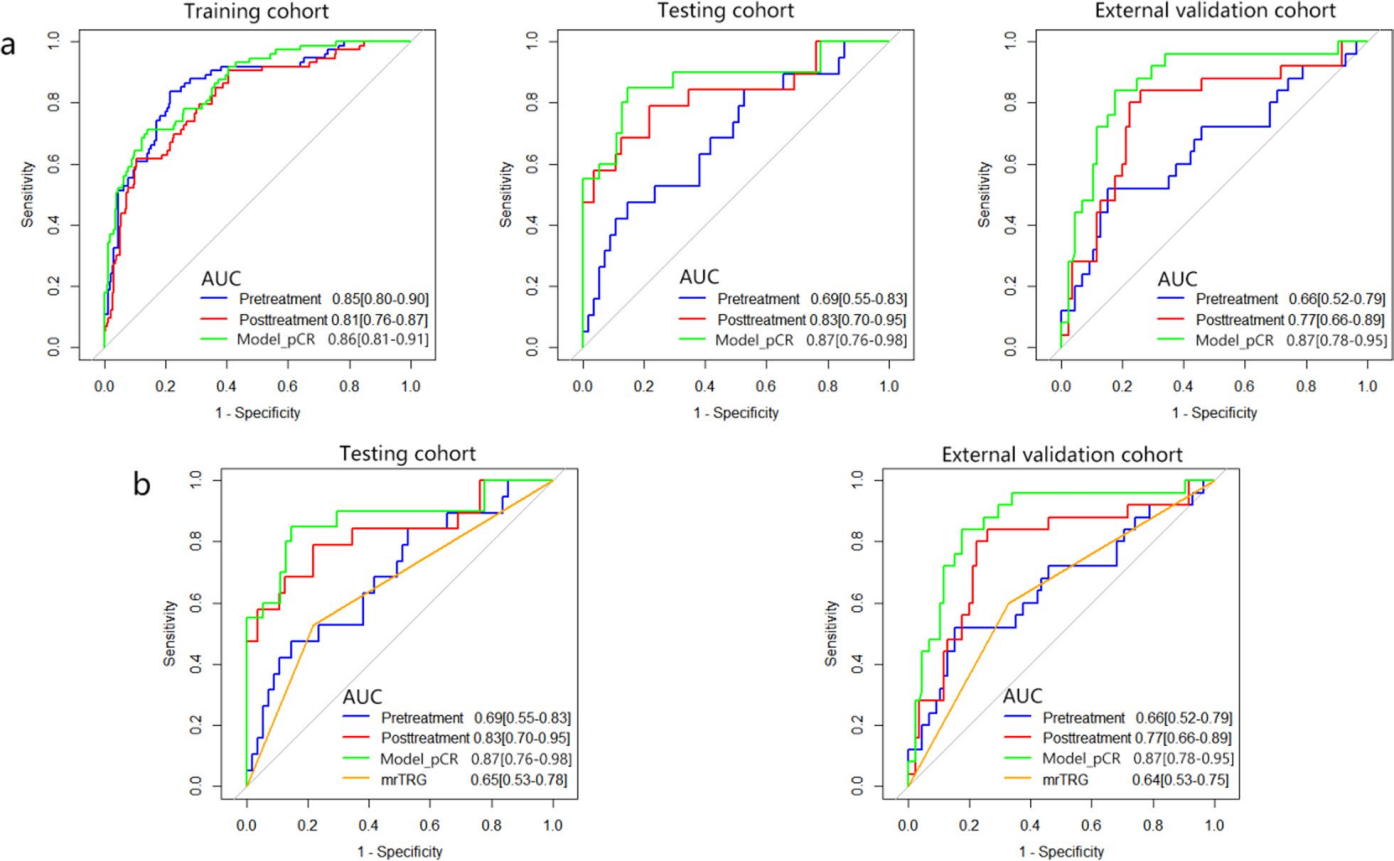
Comparison of prediction performance among MRI-based radiomics models and the mrTRG model. **a** Receiver operating characteristic curve analysis of Model_pCR compared with two single-phase mpMRI prediction models (pretreatment and posttreatment models) in the training cohort (*n* = 292), internal testing cohort (*n* = 74), and external validation cohort (*n* = 110). **b** Receiver operating characteristic curve analysis of mrTRG compared with MRI-based radiomics models in the internal testing cohort and external validation cohort. Pretreatment model, pre-NAT multiple parameter MRI-based radiomics model; posttreatment model, post-NAT multiple parameter MRI-based radiomics model; Model_pCR, multiple phase and multiple parameter MRI-based radiomics model; pCR, pathological complete response

**Fig. 4:**
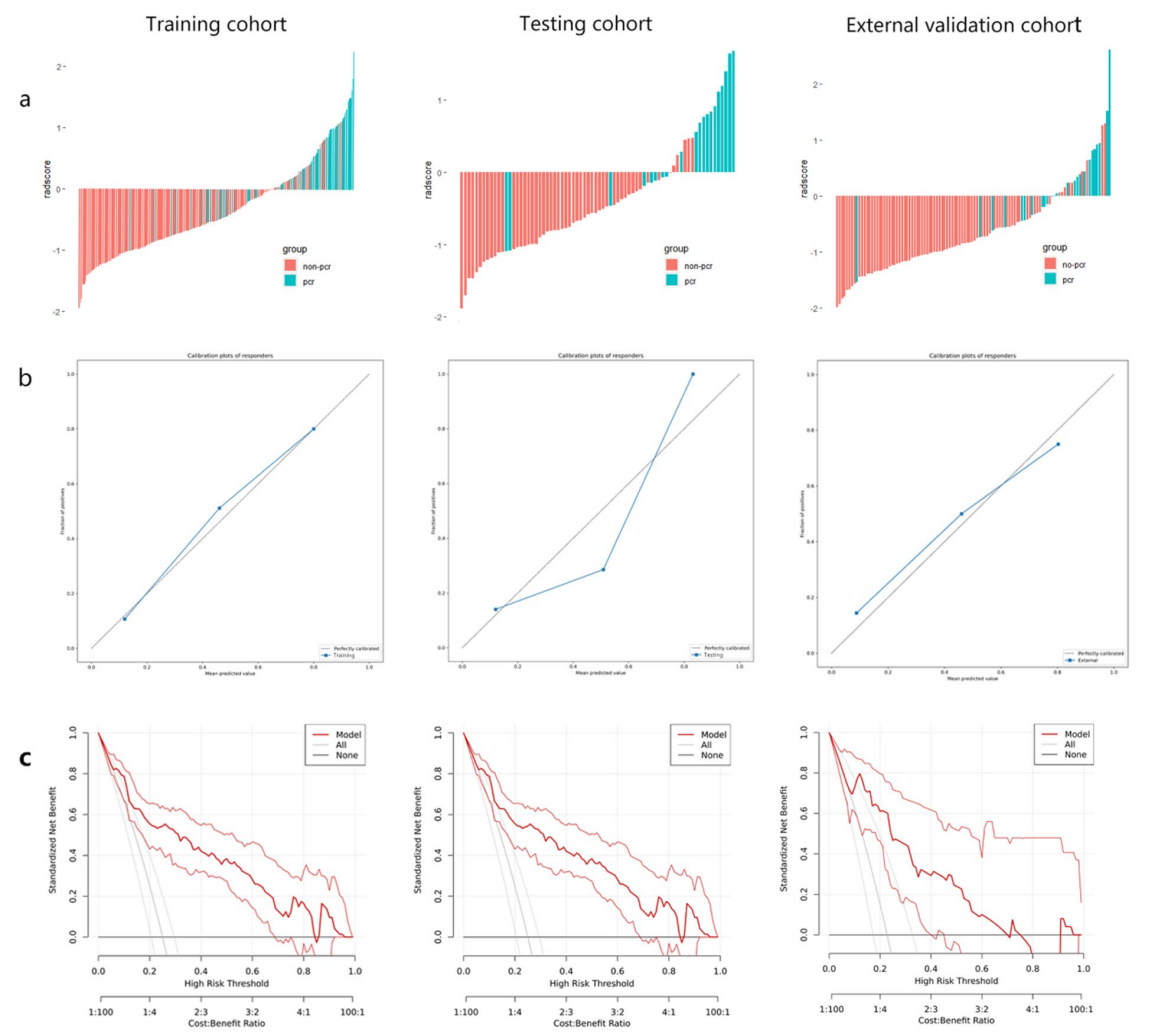
Radscore, calibration curves, and decision curves to evaluate pCR. **a** Radscore for each patient in the training cohort (*n* = 292), internal testing (*n* = 74), cohort and external validation cohort (*n* = 110). **b** Calibration curves for Model_pCR in the training cohort, internal testing cohort, and external validation cohort. The dotted reference line indicates perfect calibration. **c** Decision curves for Model_pCR in the training cohort, internal testing cohort, and external validation cohort. The y-axis represents the net benefit. The x-axis represents the threshold probability. The red line represents Model_pCR. The gray line represents the assumption that all patients achieved pCR. The black line represents the hypothesis that no patients achieved pCR. The threshold probability is where the expected benefit of treatment is equal to the expected benefit of avoiding treatment. Model_pCR, multiple phase and multiple parameter MRI-based radiomics model. pCR, pathological complete response

A total of 21 imaging features were chosen from both the pre-NAT and post-NAT mpMRI for developing Model_pCR to identify pCR in LARC patients after NAT but before surgical resection (Additional file 1: Table S7). Incorporating these features to evaluate pCR yielded favorable AUC values of 0.87 (95% CI 0.76–0.98) and 0.87 (95% CI 0.78–0.95) in the testing cohort and external validation cohort, respectively. The sensitivity and specificity of Model_pCR were excellent (84–87%). The NPV was relatively high (93.8–94.5%), while the PPV was marginally lower (56.8–65.4%) (Table 2). Model_pCR showed improved performance over single-phase mpMRI prediction models (AUC = 0.69 and 0.66 for the pre-NAT mpMRI model in the testing cohort and external validation cohort, respectively, AUC = 0.83 and 0.77 for the post-NAT mpMRI model in the testing cohort and external validation cohort, respectively) (Additional file 1: Table 8, Fig. 3a). When comparing with the mrTRG model, all three MRI-based radiomics models showed superior performance in AUC, sensitivity, specificity, and accuracy in two validation cohorts (Fig. 3b, Additional file 1: Tables S9–S10). Figure 4a shows significant differences in the radscore between pCR and non-pCR (*p* < 0.001), suggesting that Model_pCR could accurately predict pCR. Calibration curves exhibited good agreement between observations and predictions in all validation cohorts (Fig. 4b). The decision curve indicated that the multiphase and multiparameter model (Model_pCR) could bring more clinical net benefits for patients than default strategies (treat all, treat no one) (Fig. 4c).

## Discussion

In this study, with relatively large samples from two independent centers, we constructed two radiomics models to predict tumor response to NAT and to noninvasively assess pCR in LARC patients after NAT but before surgical resection. Firstly, we constructed a radiomics model for early prediction of non-response to NAT before its administration using pre-NAT mpMRI. The model showed favorable predictive performance, with an AUC of 0.81 and 0.79 in the internal testing group and independent external validation group, respectively. Secondly, we constructed a model with both pre-NAT and post-NAT mpMRI for noninvasively assessing pCR in LARC patients after NAT but before surgical resection, which exhibited excellent diagnostic performance, with an AUC of 0.87 and 0.87 in the internal testing group and independent external validation group, respectively. Moreover, the model constructed with pre-NAT mpMRI (Model_NoRes_NAT) showed better classification performance than single-sequence MRI for prediction of non-response to NAT, while the model constructed with multiphase mpMRI (Model_pCR) performed better than single-phase mpMRI and mrTRG for identification of pCR. Thus, our study provides a reliable and robust tool to identify and predict tumor response to NAT, which may assist in individualized management of LARC.

Numerous studies have attempted to identify biomarkers for early prediction of good and poor response to NAT in LARC patients before initial treatment from genetic [30] and molecular perspectives [31], but no robust predictive factors have been identified [32]. Recently, two radiomics models based on pre-treatment MRI were applied to predict non-response to NAT in LARC patients, which achieved good predictive performance, with an AUC of 0.773 [23] and 0.83 [33]. Although these studies provided important preliminary results, they also had critical limitations and thus were far from conclusive. For example, Yang et al. reported that a radiomics model based on a pre-treatment apparent diffusion coefficient image could be used to predict non-response to NAT in LARC with an AUC of 0.83 in their testing group [33]. However, they only recruited 89 LARC patients from a single center, which resulted in low robustness due to potential risks of overfitting with small sample sizes and poor generalizability due to lack of external validation [34]. Although Zhou et al. included a relatively large sample size (*n* = 425) in their study and achieved an acceptable predictive power (AUC = 0.773, 95% CI 0.608–0.937) in their testing cohort [23], their study lacked an external independent validation cohort, which limited the generalizability of their model [34]. In the current study, we recruited a large number of patients (*n* = 563) from two independent centers, although patients from two centers following different types of NAT (Center 1, 50 patients treated with neoadjuvant chemotherapy and the remain 405 patients treated with neoadjuvant chemoradiotherapy and Center 2, 110 patients treated with neoadjuvant chemoradiotherapy); based on pre-NAT mpMRI signatures, our model exhibited powerful predictive value in identifying non-responders to NAT before initial treatment, with an AUC of 0.84, 0.81, and 0.79 in the training group, internal testing group, and independent external validation group, respectively. Moreover, we revealed that the performance of the mpMRI model was better than that of any single-sequence models, which is similar to previous reports [23], it is not surprising, given that different imaging modalities have inherent strengths and limitations in reflecting tumor phenotype, microcirculation, and vascularization, and their integration might improve predictive performance [35, 36].

In addition, we constructed a multiphase and multiparameter radiomics model (model_pCR) for preoperatively assessing pCR after NAT based on pre-NAT and post-NAT MRI, which achieved excellent diagnostic performance, with an AUC of 0.87 and 0.87 in the internal testing group and independent external validation group, respectively. Currently, no standardized assessment criteria for pCR are available before surgical resection. The mrTRG is widely used in clinic to identify patients who have potentially achieved pCR after NAT [37]; however, the sensitivity and specificity were found to be only 62% (95% CI 43, 77) and 89% (95% CI 80, 94), respectively, in a recent meta-analysis [38]. Our radiomics model based on multiphase and multiparameter MR data achieved a much better diagnostic performance than the mrTRG model, with sensitivity and specificity of 84.0 [65.4–93.6] and 81.2 [71.6–88.1], respectively, which suggested that radiomics models may contribute to reducing misjudgment by providing complementary information about the tumor heterogeneity [21, 34]. Our study obtained a similar diagnostic performance in assessing pCR as previous radiomics studies [24, 39]. Shin et al. built a radiomics model based on post-NAT T2WI and DWI signatures, which reported promising results, with the best-performing models for predicting pCR being those based on T2-weighted images (sensitivity, 80.0% [95% CI 71.0, 89.1], and specificity, 68.4% [95% CI 62.4, 74.4]) and the merged model (sensitivity, 76.0% [95% CI 66.3, 85.7], and specificity, 71.4% [95% CI 65.6, 77.3]). Moreover, they revealed that both the T2 and merged models achieved significantly greater sensitivity than the radiologists did. Although they recruited a large sample cohort and constructed a well-performing model, as their study was a single-center study, the generalizability to other healthcare settings was unclear [24]. Jang et al. [40] developed an image-based deep learning model for predicting pCR in rectal cancer by using post-chemoradiotherapy magnetic resonance imaging. Although they also obtained a comparable predictive efficiency (AUC of 0.77), the “black-box” nature of deep learning, including implicit feature engineering or modeling, would hinder its application in clinical practice [41]. Moreover, we integrated pre-NAT and post-NAT MR data, which achieved better performance than the model constructed with pre-NAT (AUC = 0.69 and 0.66 in the internal testing and external validation groups, respectively) or post-NAT (AUC = 0.83 and 0.77 in the internal testing and external validation groups, respectively) MR data alone. A possible explanation is that post-NAT MRI directly reflects tumor regression and residual after treatment, while pretreatment MRI can provide comprehensive information on tumor heterogeneity, which might be associated with tumor response and prognosis [42]. Therefore, a combination of these approaches, which can comprehensively capture the tumor heterogeneity and tumor reaction to NAT, might improve predictive performance. A recent study also demonstrated that the combination of pre- and post-treatment MRI improved the performance of the radiomics model [43]. Thus, it is plausible that fusing multiphase and multiparameter information from both pre-NAT and post-NAT could facilitate a more comprehensive description of tumor characteristics and could undoubtedly help to establish a more precise prediction model.

Our study also had several limitations. First, we used a manual delineation method to perform tumor segmentation. Although satisfactory intra-observer and inter-observer reproducibility was acquired for the tumor segmentation and the extraction of imaging features (ICCs > 0.6), the manual delineation is a time-consuming and labor-intensive task. Therefore, a user-friendly and completely automated segmentation approach such as deep learning should be developed in the future. Second, non-imaging predictive markers, such as pathology and endoscopy, were not included in present study. Future studies should investigate whether incorporating clinical variables into a radiomics model would improve the predictive performance. In addition, patients from Center 1 underwent different types of NAT (50 of them treated with neoadjuvant chemotherapy and the remain 405 patients treated with neoadjuvant chemoradiotherapy), while patients from Center 2 treated with neoadjuvant chemoradiotherapy only. Different treatment strategies may have biased the results, future study should include patients with the same treatment regimen.

## Conclusion

In conclusion, based on multiphase and multiparameter MRI, we developed two radiomics models, for prediction of non-responders to NAT before its administration (Model_NoRes_NAT) and for identification of pCR before surgical resection (Model_pCR), respectively, both of which obtained excellent performance in the testing cohort and the independent external validation cohort. Additionally, the model constructed with pre-NAT multiparameter MRI showed better classification performance than single-sequence MRI for prediction of non-response to NAT, while the model constructed with multiphase multiparameter MRI also performed better than single-phase multiparameter MRI and mrTRG for identification of pCR, suggesting that the multiphase and multiparameter models may assist in individualized management of LARC.

### Supplementary Information

**Additional file 1**. Supplementary methods, tables, figures.

## Data Availability

Data are available from the corresponding author upon reasonable request.

## Abbreviations

LARC: Locally advanced rectal cancer
NAT: Neoadjuvant therapy
mpMRI: Multiparameters MRI
T2WI: T2-weighted imaging
DWI: Diffusion-weighted imaging
CE-T1WI: Contrast enhanced T1-weighted imaging
ICCs: Intraclass correlation coefficients
LASSO: Least absolute shrinkage and selection operator
mrTRG: MRI-based tumor regression grade
pCR: Pathologic complete response
AUC: Area under the curve
SVM: Support vector machine
