# Supplementary material for: Multiphase and multiparameter MRI-based radiomics for prediction of tumor response to neoadjuvant therapy in locally advanced rectal cancer
Source: Radiat Oncol. 2023 Oct 31;18:179. doi: 10.1186/s13014-023-02368-4 (PMC10619290; doi:10.1186/s13014-023-02368-4)
Supplement: Supplementary file 1 — Additional file 1. Supplementary methods, tables, figures. [file 13014_2023_2368_MOESM1_ESM.docx]

**Supplementary methods**

1. ***Patients***

We retrospectively reviewed data for 704 patients with locally advanced rectal cancer (LARC) according to the following inclusion and exclusion criteria.

Inclusion criteria were: (a) primary rectal adenocarcinoma confirmed through biopsy; (b) LARC diagnosed at pretreatment MRI (≥ T3, and/or positive lymph node); (c) completing the standard neoadjuvant therapy; (d) available pre-NAT MRI data (for Model_NoRes_NAT) or both pre-NAT and post-NAT MRI data (for Model_pCR) on a 3T MR scanner, including DWI, T2WI, and CE-T1WI.

Exclusion criteria were: (a) incomplete NAT; (b) lack of DWI or T2WI or CE-T1WI data at any phase; (d) insufficient image quality for tumor segmentation.

Finally, a total of 563 patients who fulfilled the inclusion criteria (for construction of Model_NoRes_NAT) were included in the present study; Out of the 563 patients, 476 fulfilled the inclusion criteria for construction of Model_pCR. Clinical characteristics, including age, gender, clinic T stage, N stage, pre-NAT carcinoembryonic antigen (CEA), and pre-NAT carbohydrate antigen-199 (CA199), were obtained from the medical records. The flowchart of patients’ recruitment is shown in Supplementary Figure 1.

1. ***Tumor segmentation and Intra- and inter-observer reproducibility evaluation***

The regions of interest (ROIs) were manually delineated slice-by-slice using the DWI, T2WI, and CE-T1WI data via the Darwin research platform (https://arxiv.org/abs/2009.00908), including the whole tumor but excluding perienteric fat, air, and feces, by a radiologist who was blinded to the clinical information and the histopathology results (reader 1, with 3 years of experience in rectal cancer imaging), one sample can be found in Supplementary Figure 2.To ensure the consistency of manual segmentation and feature extraction, 100 patients were selected randomly and segmented again with an interval of at least 1 month by reader 1 and another radiologist (reader 2) with 8 years of experience in a blind manner to assess inter-/intra-observer reproducibility of radiomics feature analysis by calculating the intra-class correlation coefficients (ICCs).**Supplementary Table 1: The MR scanning parameters of the multiple centers**

| Hospital | Scanner | Sequence | TR (ms) | TE (ms) | FOV (mm) | Matrix | Slice Thickness(mm) | Slice Gap(mm) |
| --- | --- | --- | --- | --- | --- | --- | --- | --- |
| Center 1 | SIEMENS 3.0T  (TrioTim) | T2WI | 3000-4000 | 82-84 | 100 | 384×230 | 3 | 3 |
|  |  | DWI | 5800 | 78 | 85 | 160×102 | 5 | 6 |
|  |  | CE-T1WI | 4 | 2 | 82 | 288×211 | 3 | 6 |
|  | GE 3.0T  (DISCOVERY) | T2WI | 4400-5600 | 86-95 | 100 | 320×256 | 3 | 3 |
|  |  | DWI | 5500-7000 | 63-67 | 80 | 128×128 | 5 | 6 |
|  |  | CE-T1WI | 4 | 2 | 80 | 288×208 | 3 | 5 |
|  | Philips 3.0T  (Achieva) | T2WI | 3100-3400 | 90 | 100 | 300×289 | 3 | 3 |
|  |  | DWI | 2105 | 51 | 70 | 124×124 | 5 | 6 |
|  |  | CE-T1WI | 6 | 3 | 90 | 268×263 | 4 | 5 |
| Center 2 | SIEMENS 3.0T  (Skyra) | T2WI | 4000 | 90 | 190 | 320×256 | 4 | 4 |
|  |  | DWI | 4300 | 78 | 200 | 140×140 | 4 | 5 |
|  |  | CE-T1WI | 4 | 2 | 100 | 288×208 | 3 | 5 |
|  | uMR 780 | T2WI | 3000 | 90 | 200 | 300×230 | 4 | 6 |
|  |  | DWI | 4000 | 70 | 220 | 128×128 | 4 | 4 |
|  |  | CE-T1WI | 4 | 2 | 110 | 288×215 | 2 | 2 |

Note: TR, repetition time; TE, echo time; FOV, field of view; T2WI, T2-weighted imaging; DWI, diffusion-weighted imaging; CE, contrast-enhanced T1-weighted imaging.

**Supplementary Table 2: Baseline characteristics of patients in the prediction of non-response to NAT**

|  |  | Center 1 | | | Center 2 | | |
| --- | --- | --- | --- | --- | --- | --- | --- |
|  |  | responders  (n=367) | non-responders  (n=86) | *P* | responders  (n=85) | non-responders  (n=25) | *P* |
| Age (years, mean ± SD) |  | 55.1±11.1 | 56.3±11.2 | 0.367 | 59.68±10.39 | 57.56±11.39 | 0.382 |
| Sex (%) | Male | 240 (65.4%) | 59 (68.6%) | 0.615 | 66 (77.6%) | 19 (76.0%) | 1.000 |
|  | Female | 127 (34.6%) | 27 (31.4%) |  | 19 (22.4%) | 6 (24.0%) |  |
| Clinical stage (%) | II | 77 (21.0%) | 16 (18.6%) | 0.267 | 9 (10.6%) | 2 (8.0%) | 0.567 |
|  | III | 255 (69.5%) | 66 (76.7%) |  | 75 (88.2%) | 22 (88%) |  |
|  | IV | 35 (9.5%) | 4 (4.7%) |  | 1 (1.2%) | 1 (4.0%) |  |
| Pretreatment T stage (%) | 2 | 7 (1.9%) | 3 (3.5%) | 0.552 | 1 (1.2%) | - | 0.612 |
|  | 3 | 221 (60.2%) | 48 (55.8%) |  | 41 (48.2%) | 10 (40.0%) |  |
|  | 4 | 139 (37.9%) | 35 (40.7%) |  | 43 (50.6%) | 15 (60.0%) |  |
| Lymph node status (%) | LN negative | 79 (21.5%) | 15 (17.4%) | 0.462 | 12 (14.1%) | 2 (8.0%) | 0.517 |
|  | LN positive | 288 (78.5%) | 71 (82.6%) |  | 73 (85.9%) | 23 (92.0%) |  |
| Location (%) | >10 cm | 47 (12.8%) | 21 (24.4%) | 0.024* | 7 (8.2%) | 3 (12.0%) | 0.278 |
|  | 5-10 cm | 195 (53.1%) | 41 (47.7%) |  | 29 (34.1%) | 12 (48.0%) |  |
|  | <5 cm | 125 (34.1%) | 24 (27.9%) |  | 49 (57.6%) | 10 (40.0%) |  |
| Pretreatment CEA (%) | <5 (normal) | 206 (56.1%) | 39 (45.3%) | 0.073 | 45 (52.9%) | 7 (28.0%) | 0.040* |
|  | ≥5(abnormal) | 161 (43.9%) | 47 (54.7%) |  | 40 (47.1%) | 18 (72.0%) |  |
| Pretreatment CA199 (%) | <39 (normal) | 317 (86.4%) | 75 (87.2%) | 0.864 | 74 (87.1%) | 19 (76.0%) | 0.211 |
|  | ≥39(abnormal) | 50 (13.6%) | 11 (12.8%) |  | 11 (12.9%) | 6 (24.0%) |  |
| Neoadjuvant therapy (%) | Neoadjuvant chemoradiotherapy | 339 (92.4%) | 64 (74.4%) | <0.001* | 85 (100.0%) | 25 (100%.0) | - |
|  | Neoadjuvant chemotherapy | 28 (7.6%) | 22 (25.6%) |  | - | - |  |

Note: Ages are shown as mean ± standard deviation, other data are numbers of patients, with percentages in parentheses. χ² or Fisher’s exact tests were used to test whether the variable composition varied significantly between patients with response and those without response to neoadjuvant therapy. * *P*<0.05, which is considered statistically significant difference.

**Supplementary Table 3: Baseline characteristics of patients in the evaluation of pCR**

|  |  | Center 1 | | | Center 2 | | |
| --- | --- | --- | --- | --- | --- | --- | --- |
|  |  | non-pCR  (n=288) | pCR  (n=88) | *P* | non-pCR  (n=85) | pCR  (n=25) | *P* |
| Age (years, mean ± SD) |  | 56.3±10.9 | 54.6±11.5 | 0.219 | 59.45±11.02 | 58.36±9.23 | 0.655 |
| Sex (%) | Male | 182 (65.5%) | 59 (67.0%) | 0.798 | 65 (76.5%) | 20 (80.0%) | 0.793 |
|  | Female | 96 (34.5%) | 29 (33.0%) |  | 20 (23.5%) | 5 (20.0%) |  |
| Clinical stage (%) | II | 46 (16.5%) | 17 (19.3%) | 0.621 | 7 (8.2%) | 4 (16.0%) | 0.467 |
|  | III | 210 (75.5%) | 62 (70.5%) |  | 76 (89.4%) | 21 (84.0%) |  |
|  | IV | 22 (8.0%) | 9 (10.2%) |  | 2 (2.4%) | - |  |
| Pretreatment T stage (%) | 2 | 8 (2.9%) | 1 (1.1%) | 0.113 | 1 (1.2%) | - |  |
|  | 3 | 168 (60.4%) | 44 (50.0%) |  | 39 (45.9%) | 12 (48.0%) | 1.000 |
|  | 4 | 102 (36.7%) | 43 (48.9%) |  | 45 (52.9%) | 13 (52.0%) |  |
| Lymph node status (%) | LN negative | 47 (16.9%) | 17 (19.3%) | 0.63 | 10 (11.8%) | 4 (16.0%) | 0.733 |
|  | LN positive | 231 (83.1%) | 71 (80.7%) |  | 75 (88.2%) | 21 (84.0%) |  |
| Location (%) | >10 cm | 46 (16.5%) | 5 (5.7%) | 0.017* | 10 (11.8%) | - | 0.201 |
|  | 5-10 cm | 146 (52.5%) | 46 (52.3%) |  | 30 (35.3%) | 11 (44.0%) |  |
|  | <5 cm | 86(31.0%) | 37 (42.0%) |  | 45 (52.9%) | 14 (56.0%) |  |
| Pretreatment CEA (%) | <5 (normal) | 142 (51.1%) | 59 (67.0%) | 0.01* | 35 (41.2%) | 17 (68.0%) | 0.023* |
|  | ≥5(abnormal) | 136 (48.9%) | 29 (33.0%) |  | 50 (58.8%) | 8 (32.0%) |  |
| Pretreatment CA199 (%) | <39 (normal) | 241(86.7%) | 78 (88.6%) | 0.717 | 70 (82.4%) | 23 (92.0%) | 0.350 |
|  | ≥39(abnormal) | 37 (13.3%) | 10 (11.4%) |  | 15 (17.6%) | 2 (8.0%) |  |
| Neoadjuvant therapy (%) | Neoadjuvant chemoradiotherapy | 238 (85.6%) | 85 (96.6%) | 0.007* | 85 (100.0%) | 25 (100.0%) | - |
|  | Neoadjuvant chemotherapy | 40 (14.4%) | 3 (3.4%) |  | - | - |  |

Note: Ages are shown as mean ± standard deviation, other data are numbers of patients, with percentages in parentheses. χ² or Fisher’s exact tests were used to test whether the variable composition varied significantly between patients with a pCR and those without a pCR. pCR, pathological complete response. * *P*<0.05, which is considered statistically significant difference.

**Supplementary Table 4: The selected features for model construction in the prediction of non-response to NAT**

| Model (N)a | Selected features | Coefficients |
| --- | --- | --- |
| T2WI model (N=7) | wavelet-LLL_glcm_MCC | -1.951 |
|  | wavelet-LHL_glszm_ZoneEntropy | 1.839 |
|  | wavelet-HHH_glcm_InverseVariance | 1.532 |
|  | exponential_glszm_SmallAreaEmphasis | -1.447 |
|  | lbp-3D-k_glrlm_RunPercentage | -1.407 |
|  | wavelet-HHH_glcm_ClusterShade | -1.061 |
|  | exponential_glszm_SmallAreaEmphasis | -1.025 |
| DWI model (N=10) | wavelet-LLH_ngtdm_Strength | -1.815 |
|  | wavelet-LLH_firstorder_Skewness | 1.741 |
|  | wavelet-HHH_glcm_MCC | -1.576 |
|  | logarithm_firstorder_10Percentile | 1.515 |
|  | logarithm_firstorder_Maximum | 1.398 |
|  | wavelet-HHH_glszm_GrayLevelNonUniformityNormalized | 1.135 |
|  | square_glszm_ZoneVariance | 1.12 |
|  | logarithm_firstorder_Kurtosis | 1.003 |
|  | wavelet-LLH_glcm_Correlation | 0.764 |
|  | wavelet-HLH_glszm_SmallAreaEmphasis | -0.679 |
| CE model (N=19) | wavelet-HHL_glcm_Correlation | 2.372 |
|  | log-sigma-3-0-mm-3D_firstorder_Skewness | 2.231 |
|  | wavelet-HLH_firstorder_Mean | -2.229 |
|  | wavelet-LHL_glcm_Idn | 1.505 |
|  | wavelet-LLH_firstorder_Median | 1.301 |
|  | wavelet-LHL_gldm_SmallDependenceLowGrayLevelEmphasis | -1.047 |
|  | wavelet-LHL_glcm_Idmn | 0.979 |
|  | wavelet-HLL_glcm_Correlation | -0.724 |
|  | wavelet-HHH_firstorder_Kurtosis | -0.426 |
|  | original_glrlm_LongRunLowGrayLevelEmphasis | 0.383 |
|  | lbp-2D_glrlm_ShortRunEmphasis | -0.112 |
|  | lbp-2D_glrlm_ShortRunHighGrayLevelEmphasis | -0.112 |
|  | lbp-2D_glrlm_ShortRunLowGrayLevelEmphasis | -0.112 |
|  | lbp-3D-m1_glrlm_ShortRunEmphasis | -0.112 |
|  | lbp-3D-m1_glrlm_ShortRunHighGrayLevelEmphasis | -0.112 |
|  | lbp-3D-m1_glrlm_ShortRunLowGrayLevelEmphasis | -0.112 |
|  | lbp-3D-m2_glrlm_ShortRunEmphasis | -0.112 |
|  | lbp-3D-m2_glrlm_ShortRunHighGrayLevelEmphasis | -0.112 |
|  | lbp-3D-m2_glrlm_ShortRunLowGrayLevelEmphasis | -0.112 |
| Model_NoRes_NAT (N=20) | wavelet-LLH_ngtdm_Strength_DWI | -1.393 |
|  | wavelet-LHL_glszm_ZoneEntropy_T2 | 1.392 |
|  | wavelet-HHL_firstorder_Median_T2 | 1.194 |
|  | wavelet-LHL_glcm_Idn_CE | 1.175 |
|  | lbp-3D-m2_firstorder_InterquartileRange_T2 | -1.173 |
|  | wavelet-HHL_glcm_MCC_T2 | -1.115 |
|  | lbp-2D_firstorder_90Percentile_T2 | 1.072 |
|  | exponential_glszm_SmallAreaEmphasis_T2 | -1.059 |
|  | wavelet-LLL_glcm_MCC_T2 | -1.043 |
|  | wavelet-LHL_firstorder_Median_T2 | 1.04 |
|  | logarithm_firstorder_10Percentile_DWI | 1.013 |
|  | wavelet-HHH_glcm_InverseVariance_T2 | 0.965 |
|  | log-sigma-3-0-mm-3D_firstorder_Skewness_CE | 0.914 |
|  | wavelet-HLL_glcm_MCC_T2 | 0.852 |
|  | wavelet-HHH_glcm_MCC_DWI | -0.843 |
|  | lbp-3D-m2_firstorder_90Percentile_DWI | 0.836 |
|  | wavelet-HHH_glcm_ClusterShade_T2 | -0.576 |
|  | wavelet-HLL_glcm_Correlation_CE | -0.573 |
|  | exponential_glszm_SmallAreaEmphasis_T2 | -0.565 |
|  | original_gldm_LowGrayLevelEmphasis_CE | 0.503 |

Note: (N)^a^ indicated the total number of features in distinct models. glcm, gray-level co-occurrence matrices; glrlm, gray-level run length matrix; glszm, gray-level size zone matrix; gldm, gray level dependence matrix; ngtdm, neighboring gray tone difference matrix.

**Supplementary Table 5: Performance of three single-sequence models** **in the prediction of non-response to NAT**

|  | T2WI model | | | DWI model | | | CE model | | |
| --- | --- | --- | --- | --- | --- | --- | --- | --- | --- |
|  | Training cohort | Testing cohort | External validation cohort | Training cohort | Testing cohort | External validation cohort | Training cohort | Testing cohort | External validation cohort |
| AUC [95%] | 0.71[0.64-0.78] | 0.72[0.59-0.85] | 0.68[0.55-0.80] | 0.70[0.63-0.77] | 0.63[0.49-0.76] | 0.61[0.49-0.74] | 0.71[0.64-0.78] | 0.69[0.53-0.85] | 0.43[0.29-0.56] |
| Sensitivity  [95%] | 63.2[51.4-73.7] | 88.2[65.7-96.7] | 70.8[50.8-85.1] | 73.5[62.0-82.6] | 88.2[65.7-96.7] | 83.3[64.2-93.3] | 61.6[50.2-72.0] | 76.5[52.7-90.4] | 58.3[38.8-75.5] |
| Specificity  [95%] | 71.8[66.4-76.6] | 50.0[38.9-61.1] | 62.8[52.2-72.3] | 61.2[55.5-66.6] | 43.2[32.6-54.6] | 44.2[34.2-54.7] | 89.5[84.7-92.9] | 70.3[59.1-79.5] | 37.2[27.8-47.8] |
| PPV [95%] | 34.1[26.4-42.8] | 28.9[18.3-42.3] | 34.7[22.9-48.7] | 30.5[24.0-37.9] | 26.3[16.7-39.0] | 29.4[19.9-41.1] | 66.2[54.3-76.3] | 37.1[23.2-53.7] | 20.6[12.7-31.6] |
| NPV [95%] | 89.4[84.8-92.7] | 94.9[83.1-98.6] | 88.5[78.2-94.3] | 90.9[86.1-94.2] | 94.1[80.9-98.4] | 90.5[77.9-96.2] | 87.5[82.5-91.2] | 92.9[83.0-97.2] | 76.2[61.5-86.5] |
| Accuracy | 0.70 | 0.57 | 0.65 | 0.64 | 0.52 | 0.53 | 0.83 | 0.71 | 0.42 |

Note: Data in parentheses are 95% CIs. AUC, area under the curve; PPV, positive predictive value; NPV, negative predictive value.

**Supplementary Table 6: Comparison of the AUC Values among the Model_NoRes_NAT and single-** **sequence models**

|  | Testing cohort | |  | External validation cohort | |
| --- | --- | --- | --- | --- | --- |
|  | AUC [95%] | *P* value in Comparison to Model_NoRes_NAT |  | AUC [95%] | *P* value in Comparison to Model_NoRes_NAT |
| T2WI model | 0.72[0.59-0.85] | 0.07 |  | 0.68[0.55-0.80] | 0.20 |
| DWI model | 0.63[0.49-0.76] | 0.03* |  | 0.61[0.49-0.74] | 0.04* |
| CE model | 0.69[0.53-0.85] | 0.09 |  | 0.43[0.29-0.56] | 0.01* |
| Model_NoRes_NAT | 0.81[0.70-0.91] | - |  | 0.79[0.67-0.91] | - |

Note: Data in parentheses are 95% CIs. T2WI, T2-weighted imaging; DWI, diffusion-weighted imaging; CE, contrast-enhanced T1-weighted imaging; Model_NoRes_NAT, pre-NAT multiparametric magnetic resonance imaging-based radiomics model; AUC, area under the curve. * *P*<0.05, which is considered statistically significant difference.

**Supplementary Table 7: The selected features for model construction in the evaluation of pCR**

| Model (N)^a^ | Selected features | Coefficients |
| --- | --- | --- |
| Pretreatment model (N=20) | exponential_glrlm_ShortRunLowGrayLevelEmphasis | 3.475 |
|  | wavelet-HLL_glszm_ZoneVariance | 3.266 |
|  | gradient_glcm_ClusterShade | 3.256 |
|  | wavelet-LHL_ngtdm_Strength | -2.611 |
|  | wavelet-HHH_glcm_Idm | -2.481 |
|  | original_firstorder_Kurtosis | 2.018 |
|  | gradient_firstorder_Minimum | -2.014 |
|  | wavelet-LHL_firstorder_Kurtosis | 1.897 |
|  | wavelet-LHL_glrlm_ShortRunLowGrayLevelEmphasis | 1.796 |
|  | wavelet-HLL_gldm_LargeDependenceLowGrayLevelEmphasis | 1.687 |
|  | wavelet-LHH_glcm_MCC | 1.639 |
|  | exponential_gldm_DependenceNonUniformity | 1.533 |
|  | wavelet-LHH_gldm_LargeDependenceLowGrayLevelEmphasis | -1.499 |
|  | wavelet-HLL_glcm_MaximumProbability | 1.435 |
|  | exponential_gldm_LowGrayLevelEmphasis | 1.352 |
|  | square_firstorder_RootMeanSquared | 1.254 |
|  | original_shape_Flatness | 1.252 |
|  | wavelet-HLL_glszm_SmallAreaLowGrayLevelEmphasis | 1.216 |
|  | wavelet-LHH_glcm | -0.955 |
|  | wavelet-HLL_glcm_ClusterProminence | -0.863 |
| Posttreatment model (N=15) | wavelet-HHL_glcm_Imc1 | -1.49 |
|  | exponential_glszm_SmallAreaEmphasis | 1.262 |
|  | wavelet-LLL_glcm_MCC | -1.018 |
|  | wavelet-HLL_ngtdm_Coarseness | 0.677 |
|  | wavelet-LLL_glcm_JointEntropy | -0.646 |
|  | log-sigma-3-0-mm-3D_glcm_Correlation | -0.601 |
|  | wavelet-HLH_glcm_Correlation | -0.594 |
|  | exponential_glcm_JointEnergy | -0.463 |
|  | squareroot_gldm_SmallDependenceLowGrayLevelEmphasis | 0.455 |
|  | wavelet-HHH_gldm_LargeDependenceLowGrayLevelEmphasis | -0.394 |
|  | log-sigma-3-0-mm-3D_glszm_ZoneEntropy | -0.37 |
|  | lbp-3D-k_firstorder_10Percentile | -0.223 |
|  | lbp-3D-k_glszm_SmallAreaLowGrayLevelEmphasis | -0.054 |
|  | lbp-3D-k_glszm_SmallAreaEmphasis | -0.054 |
|  | lbp-3D-k_glszm_SmallAreaHighGrayLevelEmphasis | -0.054 |
| Model_pCR (N=21) | Pre-square_glcm_Idn | 0.936 |
|  | wavelet-HLL_ngtdm_Coarseness | 0.896 |
|  | wavelet-HHL_glcm_Imc1 | -0.883 |
|  | Pre-gradient_firstorder_Minimum | -0.863 |
|  | wavelet-HHL_glcm_Correlation | -0.737 |
|  | square_gldm_SmallDependenceLowGrayLevelEmphasis | 0.66 |
|  | gradient_glcm_Imc1 | -0.623 |
|  | Pre-logarithm_firstorder_10Percentile | -0.606 |
|  | squareroot_glcm_Imc2 | -0.532 |
|  | square_glcm_MaximumProbability | 0.48 |
|  | wavelet-LHL_gldm_LargeDependenceHighGrayLevelEmphasis | -0.465 |
|  | gradient_gldm_LargeDependenceLowGrayLevelEmphasis | 0.449 |
|  | wavelet-LHH_firstorder_Kurtosis | -0.445 |
|  | wavelet-LLL_glcm_JointEntropy | -0.445 |
|  | logarithm_firstorder_Range | -0.43 |
|  | wavelet-LHH_firstorder_Kurtosis | -0.371 |
|  | log-sigma-3-0-mm-3D_firstorder_90Percentile | -0.362 |
|  | lbp-3D-k_firstorder_Maximum | -0.319 |
|  | log-sigma-3-0-mm-3D_glcm_Idmn | -0.157 |
|  | logarithm_glszm_ZoneEntropy | -0.149 |
|  | wavelet-HLH_glszm_ZoneEntropy | 0.138 |

Note: (N)^a^ indicated the total number of features in distinct models. glcm, gray-level co-occurrence matrices; glrlm, gray-level run length matrix; glszm, gray-level size zone matrix; gldm, gray level dependence matrix; ngtdm, neighboring gray tone difference matrix.

**Supplementary Table 8: Performance of the single-phase mpMRI prediction models in the evaluation of pCR**

|  | Pretreatment model | | | Posttreatment model | | |
| --- | --- | --- | --- | --- | --- | --- |
|  | Training cohort | Testing cohort | External validation cohort | Training cohort | Testing cohort | External validation cohort |
| AUC [95%] | 0.85[0.80-0.90] | 0.69[0.55-0.83] | 0.66[0.52-0.79] | 0.81[0.76-0.87] | 0.83[0.70-0.95] | 0.77[0.66-0.89] |
| Sensitivity [95%] | 83.8[75.3-91.5] | 47.4[27.3-68.3] | 52.0[33.5-70.0] | 61.6[50.2-72.0] | 79.0[56.7-91.5] | 84.0[65.2-94.5] |
| Specificity [95%] | 75.2[69.1-80.5] | 83.6[71.7-91.1] | 83.5[74.2-90.0] | 89.5[84.7-92.9] | 78.2[65.6-87.1] | 73.4[63.3-82.4] |
| PPV [95%] | 53.9[44.8-62.6] | 50.0[29.0-71.0] | 48.2[30.7-66.0] | 66.2[54.3-76.3] | 55.6[37.3-72.4] | 48.2[34.0-62.4] |
| NPV [95%] | 93.7[89.1-96.5] | 82.1[70.2-90.0] | 85.5[76.4-91.5] | 87.5[82.5-91.2] | 91.5[80.1-96.6] | 94.4[85.1-98.0] |
| Accuracy | 0.78 | 0.74 | 0.76 | 0.83 | 0.78 | 0.75 |

Note: Data in parentheses are 95% CIs. Pretreatment model, pre-NAT multiple parameters MRI-based radiomics model; Posttreatment model, post-NAT multiple parameters MRI-based radiomics model; AUC, area under the curve; PPV, positive predictive value; NPV, negative predictive value.

**Supplementary Table 9: Performance of mrTRG model in the evaluation of pCR**

|  | Training cohort | Testing cohort | External validation cohort |
| --- | --- | --- | --- |
| AUC (95%) | 0.64[0.58-0.71] | 0.65[0.53-0.78] | 0.64[0.53-0.75] |
| Sensitivity (95%) | 47.3[35.9-58.7] | 52.6[30.2-75.1] | 60.0[40.8-79.2] |
| Specificity (95%) | 81.7[76.5-85.8] | 78.2[67.3-89.1] | 67.1[57.1-77.1] |
| PPV (95%) | 46.7[35.4-58.0] | 45.5[24.6-66.3] | 34.9[20.6-49.1] |
| NPV (95%) | 82.0[76.9-87.1] | 82.7[72.4-93.0] | 85.1[76.5-93.6] |
| Accuracy | 0.73 | 0.72 | 0.66 |

Note: Data in parentheses are 95% CIs. AUC, area under the curve; PPV, positive predictive value; NPV, negative predictive value.

**Supplementary Table 10: Comparison of the AUC Values among the mrTRG model and MRI-based radiomics models**

|  | Testing cohort | |  | External validation cohort | |
| --- | --- | --- | --- | --- | --- |
|  | AUC [95%] | *P* value in Comparison to  mrTRG |  | AUC [95%] | *P* value in Comparison to  mrTRG |
| mrTRG | 0.65[0.53-0.78] | - |  | 0.64[0.53-0.75] | - |
| Pretreatment model | 0.69[0.55-0.83] | 0.7 |  | 0.66[0.52-0.79] | 0.81 |
| Posttreatment model | 0.83[0.70-0.95] | 0.06 |  | 0.77[0.66-0.89] | 0.08 |
| Model_pCR | 0.87[0.76-0.98] | 0.01* |  | 0.87[0.78-0.95] | 0.01* |

Note: Data in parentheses are 95% CIs. mrTRG, magnetic resonance image tumor regression grade; Pretreatment model, pre-NAT multiple parameters MRI-based radiomics model; Posttreatment model, post-NAT multiple parameters MRI-based radiomics model; Model_pCR, multiple phase and multiple parameters MRI-based radiomics model. * *P*<0.05, which is considered statistically significant difference.


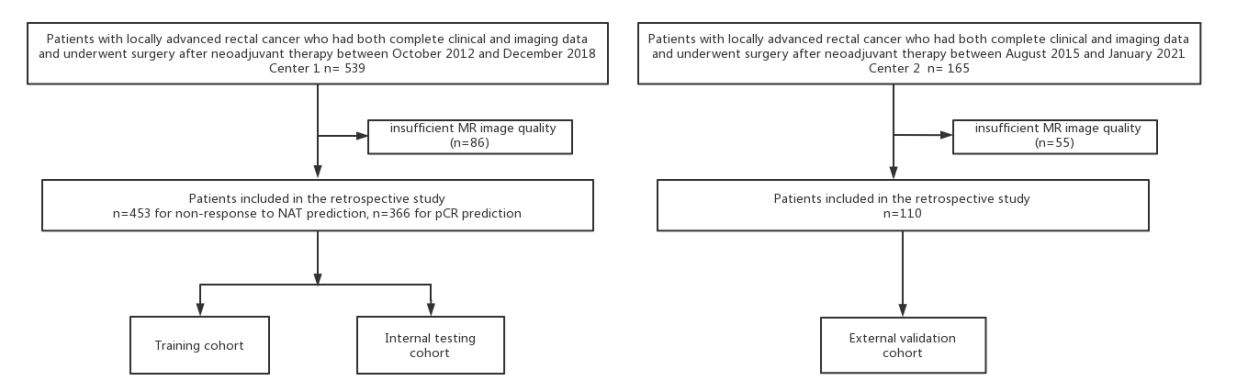


**Supplementary Figure 1: Patient recruitment and study design**

Flowchart showing patient selection and allocation to the training, testing, and external validation cohorts.


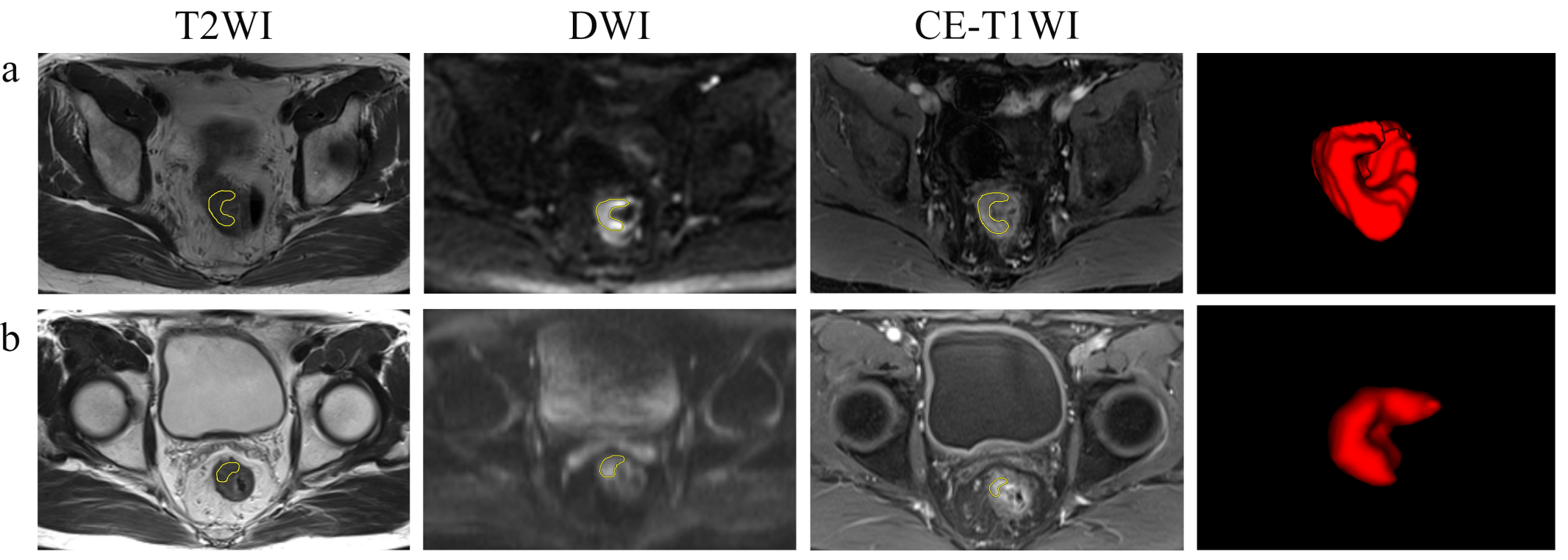


**Supplementary Figure 2: Region of interests (ROIs) in images of different sequences at the same slice**

(a) Pre-NAT MRI, (b) post-NAT MRI. Representative manual segmentation of one slice of the whole tumor in T2TI, DWI (b = 800) and CE-T1WI, respectively, and displayed as three-dimensional volumetric obtained from a 58-year-old man with rectal cancer (left to right).


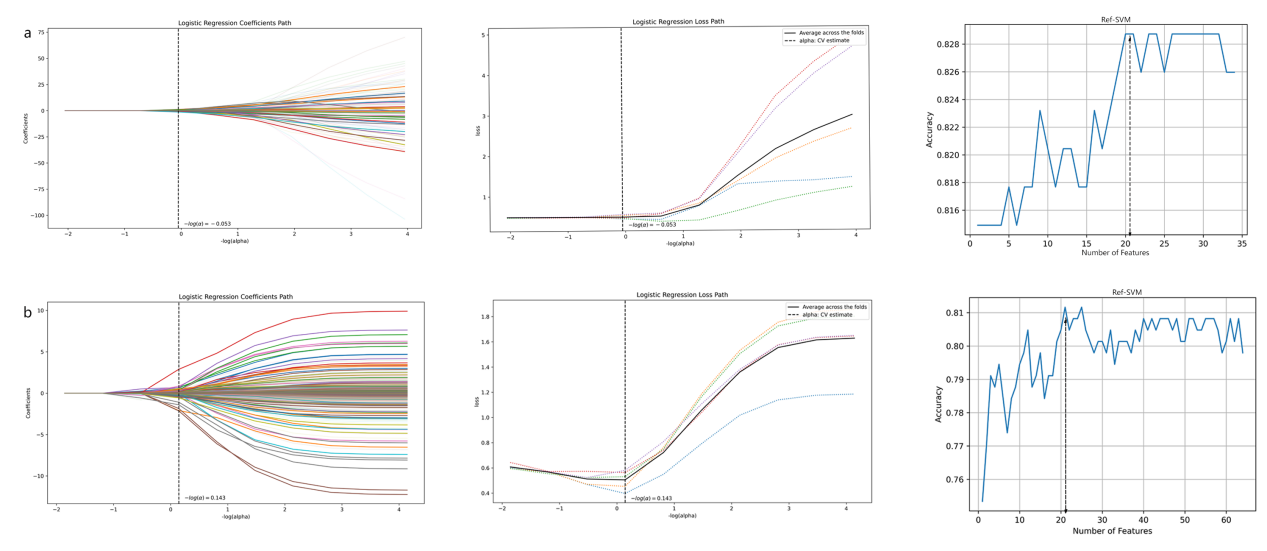


**Supplementary Figure 3: Radiomics feature selection using LASSO logistic regression and Ref-SVM**

(a, b) Significant features with non-zero weight were selected by alpha searching and an elastic network with 10-fold cross-validation for Model_NoRes_NAT and Model_pCR. Ref-SVM was applied to find the best feature combination step-by-step, and the combinations with the highest accuracy were incorporated into the models. The dotted line indicates the final selected features for model construction. LASSO, the least absolute shrinkage and selection operator; Ref-SVM, recursive feature selection support vector machine.
